# 血液肿瘤嵌合抗原受体自然杀伤细胞治疗研究进展

**DOI:** 10.3760/cma.j.issn.0253-2727.2022.12.015

**Published:** 2022-12

**Authors:** 欣笛 王, 恒 梅, 豫 胡

**Affiliations:** 华中科技大学同济医学院附属协和医院血液科，湖北省肿瘤疾病细胞治疗临床医学研究中心，武汉 430022 Institute of Hematology, Union Hospital, Tongji Medical College, Huazhong University of Science and Technology, Hubei Clinical Medical Center of Cell Therapy for Neoplastic Disease, Wuhan 430022, China

近年来，细胞治疗显现出巨大的研究潜力和临床价值。嵌合抗原受体T细胞（CAR-T细胞）治疗在血液肿瘤领域已取得重大进展，CD19 CAR-T治疗B-ALL完全缓解率高达90％[1]。但CAR-T治疗仍面临诸多挑战：①自体T细胞质量不足，异体T细胞引起移植物抗宿主病（GVHD）风险；②制备耗时；③细胞因子释放综合征（CRS）和免疫效应细胞相关神经毒性综合征（ICANS）等不良反应；④复发；⑤价格高昂。CAR-自然杀伤（NK）细胞可以在一定程度上弥补CAR-T治疗的局限，是最具潜力的新一代CAR细胞治疗产品。2020年一项靶向CD19 CAR-NK治疗B系肿瘤的临床研究实现了73％的缓解率和64％的完全缓解率，并且无GVHD、CRS和ICANS发生，首次确定CAR-NK临床应用的有效性和安全性[2]。本文我们主要对NK细胞生物学特点、CAR-NK作用机制、CAR-NK与CAR-T的比较、血液肿瘤CAR-NK研究现状、目前的优化措施进行了总结归纳。

一、NK细胞生物学特点

NK细胞属于大颗粒淋巴细胞，在外周血中占10％～15％，是天然免疫反应的关键组分[3]。作为第一道免疫防线，NK细胞可以自发杀伤肿瘤细胞而不需抗原提呈。NK细胞表面有大量的激活型受体（NKG2D、NKp46、NKp44、NKp30、CD16、2B4、DNAM1等）和抑制性受体（NKG2A、KIRs、TIGIT、CD96、LAG3、TIM3、PD1、KLRG1、CD161等），对靶细胞的耐受或杀伤取决于这两类受体信号输入的动态平衡[4]。NK细胞激活后脱颗粒，释放多种细胞毒性物质（颗粒酶B、穿孔素、粒溶素），导致靶细胞裂解。

NK细胞表面的CD16（FcγR Ⅲ）受体可以识别结合在靶细胞表面的抗体Fc段，通过抗体依赖的细胞介导的细胞毒作用（ADCC）以颗粒酶B依赖性机制发挥裂解靶细胞的功能[5]。表面的Fas配体和肿瘤坏死因子相关凋亡诱导配体（TRAIL）可以分别和肿瘤细胞表面的Fas和TRAIL受体结合，诱导靶细胞内凋亡相关蛋白酶的活化，触发靶细胞内死亡受体依赖性凋亡机制[6]。激活的NK细胞可以分泌多种趋化因子招募T细胞、单核细胞和中性粒细胞，分泌IFN-γ、TNF-α、GM-CSF和IL-10等细胞因子调节树突状细胞、巨噬细胞和T细胞，从而调动更多的免疫细胞参与抗肿瘤免疫反应[7]。

二、CAR-NK作用机制

CAR结构是基于T细胞或NK细胞活化所依赖的信号设计的，包活激活受体信号（信号1）、共刺激信号（信号2）和细胞因子信号（信号3）。CAR-NK细胞的CAR结构包含胞外段、跨膜段和胞内段，早期CAR-NK设计延用CAR-T细胞中的CAR结构。CAR-NK胞外段主要是抗体单链可变区。CAR-NK常用的跨膜段是由CD3ζ、CD8或CD28改造而来，其中T细胞特异性的CD8和CD28最常用。胞内段负责CAR-NK接收到靶抗原信号后的细胞激活，是信号传导下游所招募的共刺激分子和信号结构域的线性结构。目前CAR-NK中涉及的共刺激分子主要包括免疫球蛋白超家族成员（CD28），TNF受体超家族成员（4-1BB、CD27和OX40）, 信号通路淋巴细胞活化分子相关受体家族（2B4）。胞内段不同分子构成是区分目前四代CAR的主要分类依据。第一代CAR只包含CD3ζ，第二代和第三代则在第一代基础上分别添加一个或两个共刺激结构域，第四代则在第三代基础上增加了细胞因子分泌段，以维持CAR细胞的细胞毒性和持久性。目前CAR-NK领域最常用的为CD28-CD3ζ和41BB-CD3ζ两种二代CAR结构；而在第三代中，CD28-41BB-CD3ζ最常用[8]。另外，回输CAR-NK细胞的KIR与受者KIR配体不匹配可通过“迷失自我”机制增强NK细胞内在的非CAR依赖性抗肿瘤活性[2]。

三、CAR-NK与CAR-T治疗的比较

相比较而言，CAR-NK不仅可以弥补前述CAR-T的不足，而且显示出额外的优势（表1）。对于异体来源的细胞，异体CAR-T即使在相合情况下也有免疫排斥的风险，异体CAR-NK即使低HLA相合度（1/6）条件下也未观察到GVHD事件[2]。另外，KIR配体与受体的不匹配更能激活NK细胞天然杀伤毒性。肿瘤细胞可以通过下调主要组织相容性复合物（MHC）Ⅰ类分子逃避T细胞的识别杀伤，但是基于“迷失自我”机制NK细胞可以识别杀伤这些细胞。另外，CAR-NK联合单克隆抗体可以通过ADCC增强肿瘤清除效果，治疗策略具有多样性。在安全性方面，CAR-T细胞发挥效应过程中产生的INF-γ、TGF-α、IL-1和IL-6因子等可以引发CRS和ICANS。然而，NK细胞主要分泌IL-3和GM-CSF，引起CRS和ICANS风险极低，并且在临床试验（NCT03056339）中未观察到相应不良事件[2]。同时，由于肿瘤微环境中PD-1的表达，CAR-T细胞的功能受到抑制，而NK细胞受影响较小。CAR-NK细胞将保留其固有的通过激活性受体识别和靶向肿瘤细胞的能力，使肿瘤细胞通过下调CAR靶抗原导致复发的可能性低于CAR-T细胞[9]。多来源、多杀伤途径以及低恶性不良反应风险使CAR-NK细胞更有潜力成为通用型产品（表2）。在血液肿瘤和实体瘤临床前实验中，CAR-NK在和CAR-T的对比杀伤中呈现早期快速高效杀伤和低毒性的特点[10]–[11]。虽然可获得的CAR-NK临床数据有限，但是大量的临床前研究所显示的CAR-NK的肿瘤控制能力，展示了其未来应用于临床的美好前景。

**表1:** CAR-T与CAR-NK细胞的特征比较

细胞类型	来源	制备	杀伤毒性	不良反应	微环境抑制	复发
CAR-T	自体（患者T细胞的数量和质量问题）；异体（GVHD风险）	个体化的T细胞采集和制备耗时	CAR依赖性细胞毒性	CRS和ICANS发生率高，严重的不良反应可导致患者死亡	免疫检查点表达水平较高	多种复发机制
CAR-NK	异体原代NK细胞（外周血、脐血、干细胞）；自体原代NK细胞；NK细胞系（NK-92细胞系）	通用型，现货	CAR依赖性和非CAR依赖性细胞毒性	CRS和ICANS发生率低	免疫检查点表达水平较低	复发可能性相对较小

注 CAR-T：嵌合抗原受体T细胞；CAR-NK：嵌合抗原受体自然杀伤细胞；GVHD：移植物抗宿主病；CRS：细胞因子释放综合征；ICANS：免疫效应细胞相关神经毒性综合征

**表2:** 嵌合抗原受体自然杀伤细胞（CAR-NK）来源

细胞来源	获得性	功能表型	制备过程	细胞产品属性
PB-NK	需要供者或自体NK细胞，占外周淋巴细胞的10%~15%	成熟表型	NK细胞分选，CAR表达载体转染/转导，扩增	异质性产品
CB-NK	来自全球脐血库，数量极少，占CB淋巴细胞的15%~30%	抑制性受体NKG2A表达水平高，对K562细胞杀伤毒性弱		异质性产品
iPSC-NK	增殖能力高	不成熟表型，低CD16表达	分化，转染/转导和扩增；或转染/转导、分化和扩增	同质性产品
NK-92 ^a^	NK淋巴瘤细胞系，增殖能力高	缺乏CD16表达	转染/转导，扩增，辐射	同质性产品

注 PB-NK：外周血来源的NK细胞；CB-NK：脐血来源的NK细胞；iPSC-NK：诱导多能干细胞来源的NK细胞。^a^ NK-92细胞系，该细胞系制备和编辑容易，对冻融循环的敏感性低，临床输注前需辐照

四、血液肿瘤领域CAR-NK研究现状

截至2021年11月，在血液肿瘤领域的临床前研究有50多篇，Clinicaltrials注册的CAR-NK临床试验有20项，具体见表3。

**表3:** 2021年11月Clinicaltrials注册的嵌合抗原受体自然杀伤细胞（CAR-NK）临床研究

疾病	靶点	NK细胞来源	CAR结构（包括细胞因子）	剂量	预处理方案	治疗方案	期	NCT号	状态
难治/复发B系肿瘤	CD19	–	OX40-CD3ζ；mIL-15	3×10^8^（<50 kg者2×10^6^/kg），d 0、7、14，28 d为1周期	–	单药	1期	NCT05020678	招募
B系肿瘤	CD19	CB-NK	–；自分泌IL-15	1×10^5^/kg、1×10^6^/kg、1×10^7^/kg（±20%）	氟达拉滨、环磷酰胺	单药	1期	NCT04796675	招募
B系肿瘤	CD19	CB-NK	CD28-CD3ζ；自分泌IL-15	1×10^5^/kg、1×10^6^/kg、1×10^7^/kg	氟达拉滨、环磷酰胺、美司钠	单药	1/2期	NCT03056339	招募
B系肿瘤	CD19	–	CD28-4-1BB-CD3ζ	–	–	移植前的桥接治疗	1/2期	NCT02892695	未知
难治/复发B系淋巴瘤	CD19/22	–	–	（0.5~6）×10^5^/kg	–	单药	1期（早）	NCT03824964	未知
难治/复发B系淋巴瘤和CLL	CD19	iPSC-NK	NKG2D-2B4-CD3ζ；IL-15RF	–	氟达拉滨、环磷酰胺	单药或联合利妥昔单抗	1期	NCT04245722	招募
难治B系淋巴瘤	CD22	–	–	（0.5~6）×10^5^/kg	–	单药	1期（早）	NCT03692767	未招募
难治B系淋巴瘤	CD19	–	–	（0.5~6）×10^5^/kg	–	单药	1期（早）	NCT03690310	未招募
难治B系淋巴瘤	CD19	–	–	–	–	单药	1期（早）	NCT03824951	未知
B系淋巴瘤	CD19	CB-NK	CD28-CD3ζ；自分泌IL-15	–	卡莫司汀、阿糖孢苷、依托泊苷、马法兰、利妥昔单抗（移植前的化疗方案）	联合大剂量化疗和自体移植	1/2期	NCT03579927	撤销
自体移植后的NHL	CD19	iPSC-NK	NKG2D-2B4-CD3ζ；IL-15RF	9×10^7^、3×10^8^、9×10^8^	–	联合利妥昔单抗	1期	NCT04555811	招募
NHL	CD19	–	–	2×10^6^/kg、6×10^6^/kg、2×10^7^/kg	氟达拉滨、环磷酰胺	单药	1期（早）	NCT04639739	未招募
NHL	CD19	–	–	–	–	单药	1期	NCT04887012	招募
难治/复发ALL	CD19	PB-NK	4-1BB-CD3ζ	–	–	单药	1期	NCT00995137	完成
MM	BCMA	CB-NK	–	（1~3）×10^6^/kg、（3~6）×10^6^/kg、（0.6~1.2）×10^7^/kg	氟达拉滨、环磷酰胺	单药	1期（早）	NCT05008536	未招募
MM	BCMA	NK-92	–	–	–	单药	1/2期	NCT03940833	招募
难治/复发AML	CD33	NK-92	CD28-CD137-CD3ζ	–	–	单药	1/2期	NCT02944162	完成
AML	CD33	–	–	（1~3）×10^6^/kg、（3~6）×10^6^/kg、（0.6~1.2）×10^7^/kg	氟达拉滨、环磷酰胺	单药	1期	NCT05008575	未招募
AML、MDS	NKG2D	–	OX40-CD3ζ；mIL-15	方案A：1×10^8^（< 50 kg者2×10^6^/kg），d 0、7、14，28 d为1周期；方案B：1.5×10^8^（< 50 kg者3×10^6^/kg），d 0、7，28 d为1周期	氟达拉滨、环磷酰胺	单药	1期	NCT04623944	招募
CD7^+^白血病和淋巴瘤	CD7	–	CD28-4-1BB-CD3ζ	–	–	单药	1/2期	NCT02742727	未知

注 CLL：慢性淋巴细胞白血病；NHL：非霍奇金淋巴瘤；ALL：急性淋巴细胞白血病；MM：多发性骨髓瘤；AML：急性髓系白血病；MDS：骨髓增生异常综合征；PB-NK：外周血来源的NK细胞；CB-NK：脐血来源NK细胞；iPSC-NK：诱导多能干细胞来源NK细胞；NK-92：NK-92细胞系；mIL-15：膜表达IL-15；IL-15RF：IL-15/IL-15Rα融合蛋白；–：未提及

1. B系淋巴瘤和白血病：基于CAR-T在B系肿瘤中的成功应用，CAR-NK在B系淋巴瘤和白血病中同样取得瞩目的成绩。靶向CD19的CAR-NK治疗B系肿瘤的临床研究结果显示CAR-NK细胞治疗在患者中实现了73％的缓解率和64％的完全缓解率[2]。首次确定CAR-NK临床应用的可行性和有效性。现有的临床前和临床研究靶点主要有CD19、CD20、CD22、EBNA、WT-1和CD19/CD22双靶。在目前注册的CAR-NK临床试验中，针对B系肿瘤的占绝大多数。双靶CAR-NK的研究也率先在B系肿瘤中开展[12]。

2. 多发性骨髓瘤（MM）：在MM领域，CAR-NK研究的靶点主要有BCMA、CS1（SLAMF7）、CD138、CD19和NKG2DL。CD138 CAR-NK-92MI和CS1 CAR-NK92都呈现出良好的肿瘤控制能力并延长MM异种移植小鼠生存期[13]–[14]。除了NK细胞系的应用，自体CAR-NK也显示出优越的效果。从MM患者体内分离出激活和扩增的NK细胞，并针对MM细胞表面高表达的NKG2DL（MICA、MICA/B、ULBP-1、ULBP-2、ULBP-3等）制备NKG2D CAR-NK细胞，显示出高效的肿瘤清除率，且对正常细胞毒性低，小鼠体内也未观察到GVHD和治疗相关毒性[10]。

3. T系肿瘤：在T系肿瘤领域，目前CAR-NK的研究靶点有CD3、CD4、CD5和CD7。Chen等[15]给予3次CD3 CAR-NK92治疗显著改善了Jurkat细胞种植小鼠的生存情况，并且两周内瘤负荷降低87％[15]。CD4为成熟T细胞的标志，CD4 CAR-NK在有效杀伤肿瘤细胞的情况下不影响造血干细胞和早期祖细胞的功能，展示出较高的安全性，并且2次CAR-NK92输注延长了小鼠生存时间[16]。CAR-NK最早在T系肿瘤中应用的多次输注模式为后续CAR-NK的临床应用提供了新的思路。由于目前的T系靶点缺乏肿瘤特异性，往往引起T细胞再生障碍。基于CAR-NK治疗的安全性，研究者提出在CD3 CAR-NK治疗前/中/后联合移植降低肿瘤负荷，同时通过清除供者来源T细胞避免CVHD，通过清除宿主T细胞避免急性器官排斥反应[15]。

4. 急性髓系白血病（AML）：CAR-NK在AML领域目前的研究靶点主要有CD4、CD7、CD33、CD38、CD123、FLT3和NKG2D，由于目前的靶点在髓系细胞或其他系细胞上广谱表达，细胞治疗在AML上应用有限，更特异性的靶点目前正在探索中。CD70 KO CD70 CAR-NK [17]和CLL-1 CAR-NK联合CISH敲除的临床前数据显示靶向治疗AML有一定潜力[18]。新一代的FLT OR CD33 NOT MECN 逻辑门CAR-NK细胞研究（SENTI-202）显示，一方面激活性CAR可以识别FLT3或CD33，优于传统的单靶FLT3或CD33 CAR-NK，另一方面抑制性CAR可以保护高达67％的FLT3阳性正常细胞免受激活性CAR所介导的细胞毒性[19]。逻辑门CAR-NK的应用在治疗AML上有较大的探索空间。

五、提高CAR-NK治疗的有效性

随着CAR-NK研究的深入，研究者们采取一系列优化措施来提高CAR-NK的有效性。

（一）优化CAR结构

1. NK特异性CAR结构：NK细胞特异性的典型分子NKG2D、2B4和DNAM1被用来探索更优化的跨膜段，CD107a脱颗粒效应和细胞毒性更强[8]。另外，跨膜段设计应遵循NK细胞上跨膜蛋白的自然方向（N→C端），天然NKG2D分子具有C→N端跨膜区域，且具有短的细胞质尾，直接应用效果受限，而逆转NKG2D 跨膜段为N→C端可能增强CAR效应[11]。

研究者对CAR-NK的CAR胞内段也进行了更深入的探索。在靶向CD5的CAR-NK治疗T系肿瘤的临床前实验中，2B4ζ-CAR-NK相比于41BBζ-CAR-NK有更高水平的激活受体（CD69、NKG2D、HLA-DR）表达和更高水平的细胞因子（IFN-γ和TNF-α）分泌[20]。信号结构域除了CD3ζ，NK特异性的DAP10、DAP12也被用来进行了优化CAR结构设计的探索，但是效果欠佳。DAP12.CAR与CD3ζ.CAR的杀伤毒性无显著差异，但是CD3ζ.CAR细胞有更慢的CAR表达下调速度和较强的脱颗粒能力[21]。而在NKG2D-2B4ζ结构基础上再添加DAP10或DAP12也并没有对CAR-NK细胞功能引起差异性改变[11]。

而对于整个CAR结构，NK特异性跨膜段和共刺激分子组合NKG2D-2B4ζ，相较于T细胞特异性CAR CD28-41BBζ，诱导CAR-NK的CD107a表达和IFN-γ分泌水平更高，并介导细胞内下游磷酸化分子水平升高提前[11]。因此，NK细胞特异性结构域参与的不同排列组合在探索更优CAR结构中将有更大的潜能。

2. 双靶CAR结构：单靶CAR细胞可能由于靶抗原下调或阴性而导致肿瘤细胞逃逸，而表达双CAR的细胞可以提高CAR细胞的识别和靶向攻击效率。同时表达并联双靶CD19/CD22的CAR-NK细胞可以高效杀伤CD19单表达和CD22单表达的RS4;11细胞，从而避免因一种抗原丢失导致的CAR-NK失效[12]。

（二）增强细胞毒性

1. 诱导型MyD88/CD40共刺激分子（iMC）：iMC是一种核糖核酸调节的蛋白开关，在CAR-T中可以作为细胞激活剂促进CAR-T细胞增殖和改善持久性，应用到CAR-NK细胞中，研究者发现iMC激活的CAR-NK显示出细胞因子和趋化因子分泌增加，穿孔素和颗粒酶B脱颗粒水平提高，进而增强抗肿瘤细胞毒性[22]。

2. 记忆NK分化：IL-12、IL-15和IL-18预处理NK细胞可以诱导记忆表型，即细胞因子诱导的记忆样NK（ML NK）细胞。ML NK呈现出糖酵解代谢谱[23]，激活受体表达增加，并且不受KIR-KIRL相互作用的干扰，在NSG异种移植小鼠模型体内显示出更长的存活时间，并显著改善了针对一系列靶点的效应功能[24]。ML NK分化表型和CAR工程化协同增强NK细胞对耐药的白血病/淋巴瘤的反应性，并且体内实验显示19-CAR-ML NK可以降低人源化小鼠的瘤负荷并改善小鼠生存[25]。

3. 增强ADCC作用：NK-92细胞系不表达CD16分子，通过修饰编辑使其表达IgG Fc受体的高亲和力变异体（FcγRⅢ）同时联合利妥昔单抗可以增强ADCC作用[26]。同时表达高亲和力非裂解性CD16的CAR-iPSC-NK（FT596）联合利妥昔单抗目前也已用于临床试验中（NCT04245722）。

（三）延长持久性

1. IL-15：IL-15在NK的生存、发展、激活、代谢改变方面发挥重要作用，与CAR-NK细胞在体内的存续性密切相关。目前的CAR-NK产品多为自分泌IL-15，自分泌模式可以使CAR-NK持续处于高浓度IL-15微环境中，延长CAR-NK细胞的体内存续时间长达12个月[2]。另外，膜表达IL-15（mIL-15）配体可以通过细胞间交互介导的反式信号传递模式来传递更强的IL-15刺激信号，并应用于临床试验中（NCT04623944）。表达mIL-15/IL-15Rα融合蛋白的CAR-NK（FT596）产品目前也应用于治疗B系血液肿瘤的临床试验（NCT04555811和NCT04245722）。细胞因子诱导型含SH2蛋白（CIS）是IL-15信号的关键的负性调节因子，CRISPR/Cas9基因编辑敲除CISH基因的CAR-NK细胞通过增强Akt/mTORC1轴和c-MYC信号，提高有氧糖酵解水平，进而增强CAR-NK细胞效应功能[27]。

2. 多次输注：基于目前的临床和临床前数据来看，CAR-NK在体内的扩增和持续有限，单次输注虽然有很强的肿瘤杀伤效果，但是可能不能完全清除肿瘤细胞，因此连续重复输注大剂量CAR-NK细胞以维持体内CAR-NK动力学稳定和持久可能是未来CAR-NK治疗的策略[28]。

（四）减少耗竭信号

NK细胞受免疫检查点的影响相对T细胞较小，但是其在NK细胞耗竭过程中仍发挥着重要作用。PD-1在活化和更具反应性表型的NK细胞上的表达更为丰富。PD-L1阻断剂可以提高NK细胞的体内持久性和保留其细胞毒性，同时提高体内NK细胞通过ADCC的抗肿瘤效应[29]。另外，抗PD-L1抗体作为PD-L1+NK细胞的功能性激活抗原，通过p38信号通路提高NK细胞对白血病细胞的杀伤毒性[30]。TIGIT是表达在T细胞和NK细胞上的与耗竭相关的免疫检查点，高表达TIGIT的NK细胞伴随着CD107a、IFN-γ和TNF-α等活化因子的分泌减少，并与疾病进展和免疫逃逸相关[31]。研究显示离体的NK细胞IL-15活化后伴随有TIGIT表达上调，联合抗TIGIT抗体显著提高NK的肿瘤杀伤毒性[32]。阻断TIGIT可以延缓NK细胞耗竭，并在多种小鼠荷瘤模型中促进了NK细胞依赖性肿瘤免疫，并以NK依赖性方式促进了肿瘤特异性T细胞免疫[33]。

目前，血液肿瘤领域尚无CAR-NK联用免疫检查点阻断的应用，在实体瘤领域，靶向PD-L1的CAR-NK细胞（PD-L1 CAR t-haNK细胞）联合使用抗PD-1抗体和N803呈现出良好的肿瘤控制效果（NCT04847466）[34]。CAR-NK联合免疫检查点阻断的应用尚待进一步探索。

（五）避免自绞杀

CD38是MM已确立的免疫治疗靶点，而其是否可以作为AML的治疗靶点尚待探索。但是由于CD38在NK细胞上的基础表达以及离体培养过程中进一步诱导上调成为设计靶向CD38的CAR-NK的障碍。通过敲除NK细胞的CD38制备的CD38 KO CD38 CAR-NK显示出自绞杀减低和靶向攻击AML原代细胞的能力增强的现象[35]。

（六）改善趋化性

CAR-NK可以趋向于肿瘤病变部位。回输到淋巴瘤患者体内的CAR-NK淋巴结部位数目显著高于骨髓和外周血[2]。然而，体外培养的NK细胞会下调CXCR4、上调CXCR3表达，导致NK细胞骨髓归巢能力下降，而趋向于炎症部位，使NK细胞滞留在髓外组织[36]。共表达CXCR4的CAR-NK向骨髓巢的迁移能力增强，并显著降低MM移植小鼠模型股骨区域的瘤负荷[21]。

六、CAR-NK治疗的安全性

NK细胞表面抑制性受体识别正常细胞表面的MHC Ⅰ类分子通过“耐受”机制来抑制对正常细胞的毒性。异体输注时也不会介导GVHD的发生。对于工程化的CAR-NK，为了避免和抵消增强疗效相关措施带来的毒性风险，相关“自杀”开关也同时被设计于CAR-NK中。通过加入一个正交调节的促凋亡开关，即雷帕霉素诱导的Caspase-9（iRC9），iRC9二聚化促进CAR-NK细胞凋亡，表现为雷帕霉素呈剂量依赖性的诱导细胞表面Annexin Ⅴ表达[22]。在已发表的临床试验中，CAR-NK显示出较高的安全性，低GVHD、CRS和ICANS风险，研究者并没有激活iCR9开关[2]。

七、结语

CAR-NK在疗效方面显示出巨大的潜能，在安全性方面显示出巨大的优越性。但是CAR-NK也存在大规模制备方法改进、冻存措施优化和疗效增强的需求，同时体内存续时间短、耗竭问题尚待解决。总体来说，CAR-NK在未来更有可能成为通用型细胞产品，在单药或者联合移植、单抗等治疗方面有较大的优势。CAR-NK研究开创了细胞治疗的新思路，相信在不久的将来随着CAR-NK细胞治疗技术的成熟将给更多肿瘤患者带来福音，使人类向攻克难治复发性肿瘤治疗的难题更进一步。
